# Treatment of Epilepsy

**Published:** 1848-05

**Authors:** 


*Treatment of Epilepsy.*—M. Plouvier, of Lisle, has presented a memoir on this disease, recommending a system of treatment, which consists in the folllowing processes:

1. The exhibition of medicines which have the power of modifying the cerebral functions. Of these he prefers the sub joined combination:

| | |
|---|---|
| Extract of belladonna | grs. xxx; |
| Powdered digitalis | grs. xlv; |
| Indigo | ʒiiss; |
| Mucilage sufficient to make 50 pills. | |

Three or four days before the expected attack, he commences with one pill twice or three times a day, increasing the dose, if no effect is produced, until some degree of intoxication or somnolence declares itself. He then omits medicine altogether for two or three days after the attack and recommences as before. This plan is persisted in for a year or more, if necessary.

2. Cold baths. These are to be taken every day for two or three minutes. After coming out of the bath, the patient is to be enveloped in blankets to induce perspiration.

3. The boot of Junot. This is an apparatus by which great determination of blood may be induced over a large space, as both or one lower extremity, upon the principle of the cupping-glass. The apparatus is made in the shape of a boot, whence its name, and the contained air is exhausted by means of an air-pump.—*Gazette Médicale.—Ranking's Abstract.*

